# Association between psychological well-being, sleep quality, and academic performance among physical therapy students

**DOI:** 10.3389/fmed.2026.1704952

**Published:** 2026-02-10

**Authors:** Noha F. Mahmoud, Leena Alghamdi, Tarfa Almuaither, Riyam Alqabani, Danah Alabdulathim, Munirah Alotaibi, Afrah Almuwais, Azza M. Atya

**Affiliations:** Department of Rehabilitation Sciences, College of Health and Rehabilitation Sciences, Princess Nourah bint Abdulrahman University, Riyadh, Saudi Arabia

**Keywords:** academic performance, flourishing, physiotherapy students, sleep quality, well-being

## Abstract

**Background:**

Sleep, a core health behavior, contributes significantly to psychological well-being, whereas flourishing represents an individual’s highest level of mental functioning and emotional health. Although both constructs are important to students’ well-being and academic outcomes, their relationship remains insufficiently investigated among physiotherapy students in Saudi Arabia.

**Aim:**

This study examined sleep quality, flourishing, and their association with academic performance in undergraduate physiotherapy students.

**Methods:**

A descriptive cross-sectional study was conducted among 248 physiotherapy students. Participants completed a sociodemographic form, the Pittsburgh Sleep Quality Index (PSQI), and the Flourishing Scale (FS). Academic performance was measured using self-reported GPA. Data were analyzed using chi-square tests, Spearman’s correlation, and ordinal logistic regression.

**Results:**

Females comprised 79.4% of the sample, with GPA distribution differing significantly by gender (*p* < 0.001). Overall, 70.6% of students reported poor sleep quality. Flourishing scores were moderate (mean = 41.5 ± 8.9) and did not differ significantly between universities (*p* = 0.124). Correlation analysis showed that sleep efficiency was positively associated with GPA (*r* = 0.150, *p* < 0.05), while use of sleep medication was negatively associated (*r* = −0.130, *p* < 0.05). Flourishing scores correlated negatively with PSQI daytime dysfunction (*r* = −0.409, *p* < 0.001) and global PSQI scores (*r* = −0.327, *p* < 0.001). After adjusting for gender, study site, and academic level, regression analysis identified shorter sleep duration (OR = 0.72, *p* = 0.011) and frequent sleep medication use (OR = 1.48, *p* = 0.035) as significant predictors of lower GPA. Male gender was also associated with lower GPA (OR = 4.31, *p* < 0.001), though this may reflect institutional differences given the gender-university confounding.

**Conclusion:**

Poor sleep quality was highly prevalent among physiotherapy students, while flourishing levels remained generally high but unrelated to academic performance. After adjusting for gender and institutional factors, regression analysis demonstrated that sleep duration and frequent use of sleep medication were significant predictors of lower GPA. These findings suggest that sleep health may be associated with academic outcomes among physiotherapy students. Future studies are needed to examine whether sleep-hygiene interventions could support academic performance in this population.

## 1 Introduction

### 1.1 Background on sleep and its physiological/cognitive importance

Sleep is a core health behavior essential for overall well-being and functioning (1). Sleep affects vital signs and brain wave activity (2, 3). Inadequate sleep may cause whole-day exhaustion and fatigue, which may lead to performance errors, some of which may be critical, as in the case of medical students (4, 5).

### 1.2 Sleep problems among university and medical students

Sleep is one of the habits and life behaviors that college students change upon entering university. Poor sleep quality can affect human well-being and is associated with mental disorders such as depression (1, 6). The new university environment brings new challenges to students on personal, social, and academic levels that negatively or positively affect their health, behavior, social relations, performance, and, hence, the quality of their well-being (7). Stress due to academic work and concerns about the future have significantly affected university students’ sleep quality, causing attention deficits and poor academic performance (1, 8). The recommended sleep duration for young adults is approximately 7–9 h of sleep each day (9). Poor sleep quality was reported among medical students; presented with short duration, late onset of sleep, and day napping (4). Poor sleep quality causes a reduction in the quality of life and workplace performance, impairment of thinking and memory, anxiety, and depression (9).

### 1.3 Relationship between sleep, psychological well-being, and flourishing

The psychological well-being concept is affected by the physical health, psychological status, beliefs, and social relationships of the person, in addition to their relationship with basic environmental features (10). Flourishing is related to mental health and includes both feeling good and getting things done. Flourishing implies that life is progressing well (11). Long ago, research and practice on mental health pathology focused on and assumed that well-being would be achieved in the absence of pathology. It is well established that a high level of well-being is beneficial for every person and society (10).

Sleep quality and quantity are important for both physical and psychological well-being. Sleep is fundamental to memory integration and consolidation, problem-solving, and many other cognitive functions related to education (5, 12). Recently, the awareness of the association between sleep quality and academic performance has increased (9). The magnitude of the current sleep problem was clarified by a number of studies that demonstrated how sleep quality was affected in the university student population (1, 4, 12–14). Altered sleep quality has been reported more than twice as often in university students as in the general population (13). Academic achievement is influenced by both sleep quality and daytime sleepiness in medical students (15). Sleep quality and academic performance can affect students’ mental well-being (16). This is especially true for medical students because of their negative impact on quality of life, cognitive performance, and other associated health conditions. However, this can be solved by careful analysis of the problem (13).

### 1.4 Research gap and study aim

The cultural and regional variation in sleep patterns underscores the importance of studying sleep behaviors within specific populations. While extensive research has examined sleep quality among medical students, comparatively fewer studies have focused specifically on physical therapy students. Recent research in this population has identified an association between sleep quality and academic outcomes, (17–20) highlighting the need for population-specific investigation, particularly in understudied regions such as Saudi Arabia. Sleep disorders differ according to race and cultural affiliation; a difference was also observed between sexes (1). Few studies in Saudi Arabia have assessed sleep quality and sleep problems of medical students (4, 5, 9, 13, 15, 16, 21). To the best of our knowledge, no study has been performed in Saudi Arabia to evaluate sleep quality, mental well-being, and academic performance among physical therapist students. The current study aimed to investigate sleep quality, mental well-being (flourishing), the association between these variables, and their relationship with academic performance among physical therapy students in Riyadh city, Saudi Arabia.

## 2 Materials and methods

### 2.1 Study design and participants

This cross-sectional study aimed to assess the association between psychological well-being, sleep quality, and academic performance among students. Specifically, the study evaluated the relationships between the Flourishing Scale (FS), Pittsburgh Sleep Quality Index (PSQI), and academic performance as measured by grade point average (GPA). The data were collected from two universities using a standardized electronic questionnaire. The study followed the ethical research principles of the WMA Declaration of Helsinki for human subjects. It was approved by the Institutional Review Board (IRB) of Princess Nourah Bint Abdul Rahman University (IRB No. H-01-R-059).

The data were collected during the academic year of 2023. Convenience sampling was used; where all physical therapy students in Riyadh city universities were encouraged to participate in the study. The total number of male and female physiotherapy students was calculated as 323 (88 males and 235 females). All physical therapy students, aged 19 years or older, enrolled at level three or higher, could take part in the study. Students in their first year (foundation year), those who disagreed to participate or sign the consent form, and those who had been diagnosed with a chronic disease or mental disorder were not allowed to participate in the study. Incomplete or invalid responses were excluded from the analysis.

### 2.2 Procedure

#### 2.2.1 Measures

The study utilized two key questionnaires: the Flourishing Scale (FS) and the Pittsburgh Sleep Quality Index (PSQI). Respondents’ rating on the Flourishing Scale indicate how well they are doing psychologically in terms of things like relationships, self-esteem, optimism, and purpose. The FS is a valid and reliable scale with a total score ranging from 8 to 56, with higher scores indicating greater levels of well-being and flourishing (22). The Pittsburgh Sleep Quality Index (PSQI) is a valid and reliable self-rating questionnaire for different types of population samples (23). It’s a widely recognized tool for assessing sleep quality. It includes seven components: subjective sleep quality, sleep latency, sleep duration, habitual sleep efficiency, sleep disturbances, use of sleep medication, and daytime dysfunction. Each component is scored on a scale from 0 (no difficulty) to 3 (severe difficulty). Daytime dysfunction is scored by adding the values for trouble staying awake and difficulty keeping up enthusiasm (each scored 0–3). The summed score is then converted to a component score from 0 to 3, with higher values indicating more daytime impairment. The component scores are then summed to produce a global PSQI score, which can range from 0 to 21. A higher global PSQI score indicates poorer sleep quality. This scoring system allows for a detailed assessment of various aspects of sleep quality, making it possible to analyze how different sleep-related issues may correlate with psychological well-being and academic performance. The Arabic version of the PSQI, validated by Suleiman et al. in Arab populations with demonstrated reliability and validity, was used in this study (24). A global PSQI score of >5 indicates poor sleep quality (23).

A personal information questionnaire prepared by the researchers was used to determine the participants’ demographic characteristics. It contained four questions: gender, university, academic level, and GPA. Academic performance was presented as a self-reported cumulative grade point average (GPA) out of five. While official GPA verification from institutional records was not feasible due to privacy constraints, students were assured of complete confidentiality and anonymity to encourage honest reporting and minimize social desirability bias. GPA was analyzed as an independent variable in the study. It was classified into four categories: poor (less than 2.5), average (2.5–3.49), good (3.5–4.49), and excellent (above).

#### 2.2.2 Statistical analysis

All statistical analyses were conducted using R software. The Shapiro-Wilk test was used to assess the normality of the included variables. Descriptive statistics were used to summarize the study variables. Continuous variables were presented as means with standard deviations for normally distributed data and as medians with interquartile ranges for non-normally distributed data. Categorical variables were summarized using frequencies and percentages. The reliability of both the Flourishing Scale and the Pittsburgh Sleep Quality Index (PSQI) was assessed using Cronbach’s alpha, a measure of internal consistency. Cronbach’s alpha values range from 0 to 1, with higher values (>0.7) indicating greater reliability and interitem correlation (25). To assess the associations between the Flourishing Scale, PSQI, and academic performance (GPA), Spearman’s rank correlation was employed due to the ordinal nature of GPA categories and non-normal distribution of several PSQI components. This non-parametric method was selected due to the ordinal nature of the GPA categories and the non-linear relationships of several PSQI components. In addition to the overall PSQI score, correlations between individual PSQI components, GPA, and the Flourishing Scale were also analyzed. Ordinal logistic regression was performed with GPA as the outcome variable. Gender was included in the regression model as a control variable to adjust for potential confounding, not as a modifiable predictor of academic performance. Given the unequal gender distribution across universities, the initial model included all clinically relevant predictors: seven PSQI components (subjective sleep quality, sleep latency, sleep duration, sleep efficiency, sleep disturbances, sleep medication use, and daytime dysfunction), Flourishing Scale score, gender, university affiliation, and academic level. Backward stepwise elimination using the Akaike Information Criterion (AIC) was applied to identify the most parsimonious model. Variables were sequentially removed if their exclusion improved (reduced) the AIC value. The proportional odds assumption was tested to confirm the validity of the model. Hypothesis testing was performed at a 5% level of significance.

## 3 Results

As presented in Table 1, the study sample included 248 students with a response rate of 76.8%. Gender distribution showed a significant difference between the two universities (χ^2^ test *p* < 0.001) The sample from University A had a mixed-gender population with 54.5% female (61) and 45.5% male (51) students. At University B, the sample consisted entirely of female students (136, 100%). GPA distribution also differed significantly between the two universities (χ^2^ test *p* < 0.001). University B had a higher proportion of students in the top GPA category (>4.5) at 63.2% compared to University A’s 43.8%. Conversely, University A had a higher percentage of students in the lower GPA categories, particularly in the 2.5–3.49 range (χ^2^ test, 11.6% vs. 0.74% at University B). The total score for the Flourishing Scale was not significantly different between University A (42.5 ± 8.75) and university B (*n* = 112, 42.5 ± 8.75 vs. *n* = 136, 40.7 ± 9.10, *t*-test, *p* = 0.124). The Global PSQI scores were not significantly different between the two universities (University A: *n* = 112, 7.47 ± 3.22; University B: *n* = 136, 7.76 ± 3.45; *t*-test, *p* = 0.493). Across both universities, a substantial majority of students (70.6%) experienced poor sleep quality. Only 29.4% of the total sample reported good sleep quality. GPA was significantly associated with gender (χ^2^ test, *p* < 0.001).

**TABLE 1:** Descriptive statistics for the study sample.

Sample characteristics and outcome measures	[ALL] *N* = 248	University A *N* = 112	University B *N* = 136	p.overall	Male	Female	p.overall
Gender:	197 (79.4%)	61 (54.5%)	136 (100%)	<0.001			
Female							
Male	51 (20.6%)	51 (45.5%)	0 (0.00%)				
GPA:				<0.001			<0.001
>4.5	135 (54.4%)	49 (43.8%)	86 (63.2%)		121 (61.4%)	14 (27.5%)	
3.5–4.49	99 (39.9%)	50 (44.6%)	49 (36.0%)		72 (36.5%)	27 (52.9%)	
2.5–3.49	14 (5.65%)	13 (11.6%)	1 (0.74%)		4 (2.03%)	10 (19.6%)	
F scale	41.5 (8.97)	42.5 (8.75)	40.7 (9.10)	0.124	41.2 (8.91)	42.8 (9.14)	0.244
Global PSQI score	7.63 (3.34)	7.47 (3.22)	7.76 (3.45)	0.493	7.82 (3.37)	6.90 (3.18)	0.073
Sleep quality				0.478			0.122
Good	73 (29.4%)	36 (32.1%)	37 (27.2%)		53 (26.9%)	20 (39.2%)	
Poor	175 (70.6%)	76 (67.9%)	99 (72.8%)		144 (73.1%)	31 (60.8%)	

Data were summarized using counts and percentages for categorical variables or mean (SD) for continuous variables. Analysis was performed using Chi-square test of independence or unpaired *t*-test for categorial and continuous variables, respectively. F scale, Flourishing Scale.

### 3.1 Descriptive statistics for the PSQI and Flourishing Scale dimensions

**FIGURE 1:**
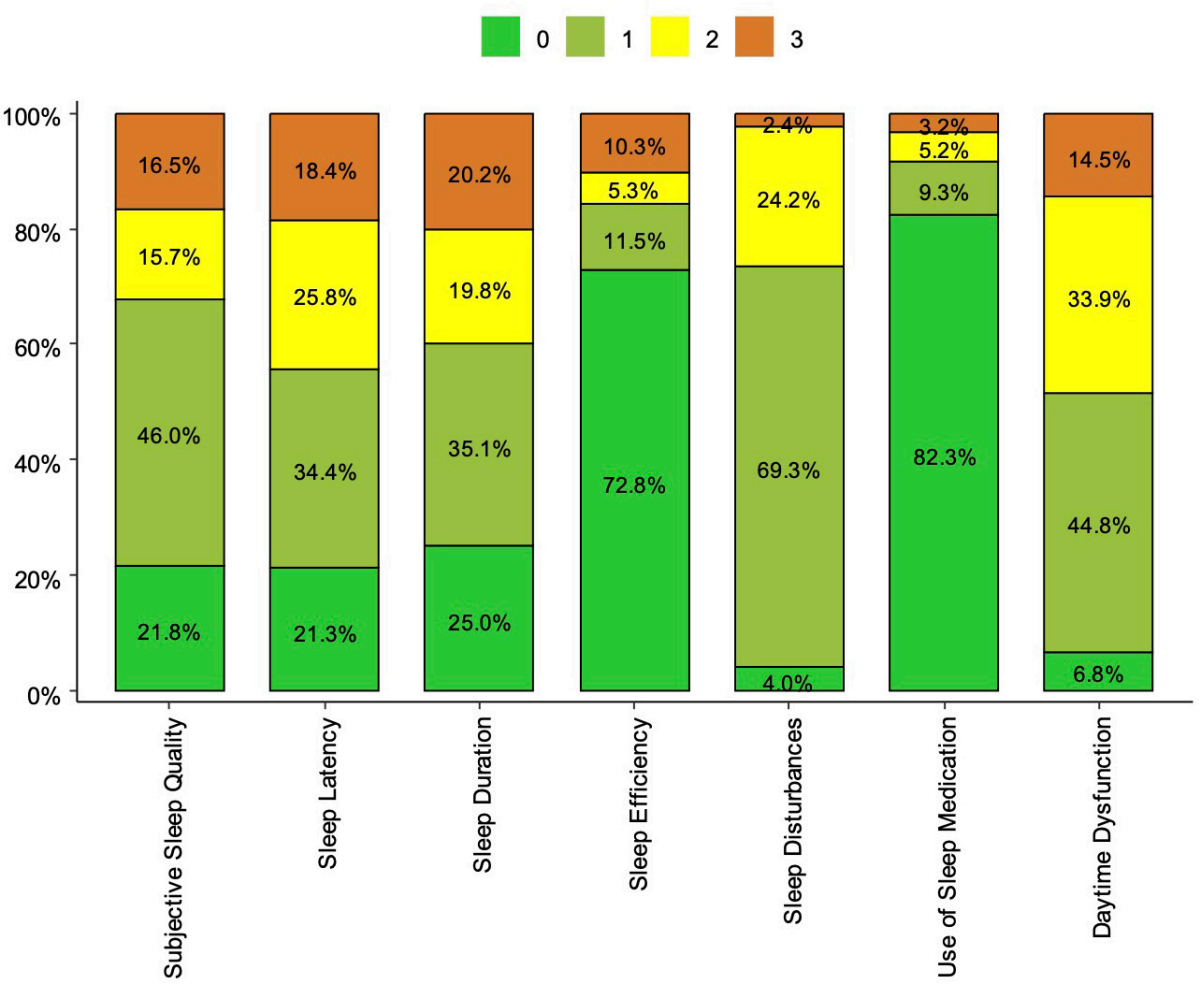
Descriptive statistics for the PSQI components.

The Pittsburgh Sleep Quality Index (PSQI) showed acceptable internal consistency (α = 0.71, 95% CI [0.61, 0.81]), while the Flourishing Scale demonstrated good reliability (α = 0.88, 95% CI [0.86, 0.90]). Daytime dysfunction was prevalent, as 93.2% of respondents reported variant levels of impairment and 14.5% experienced severe dysfunction. Sleep disturbances affected 96% of the sample, with most (69.3%) reporting mild disturbances. Sleep efficiency was high in 72.8% of respondents. However, only 25% reported optimal sleep duration, while 40% experienced poor to very poor sleep duration. Sleep latency was good to fair in 55.7%, but 44.2% struggled with poor to very poor latency. Subjective sleep quality was rated as good to fair by 67.8%, while 32.2% perceived their sleep quality as poor to very poor. Use of sleep medication occurred in 17.7% of respondents. Figure 1 illustrates the descriptive statistics for the PSQI components.

**FIGURE 2:**
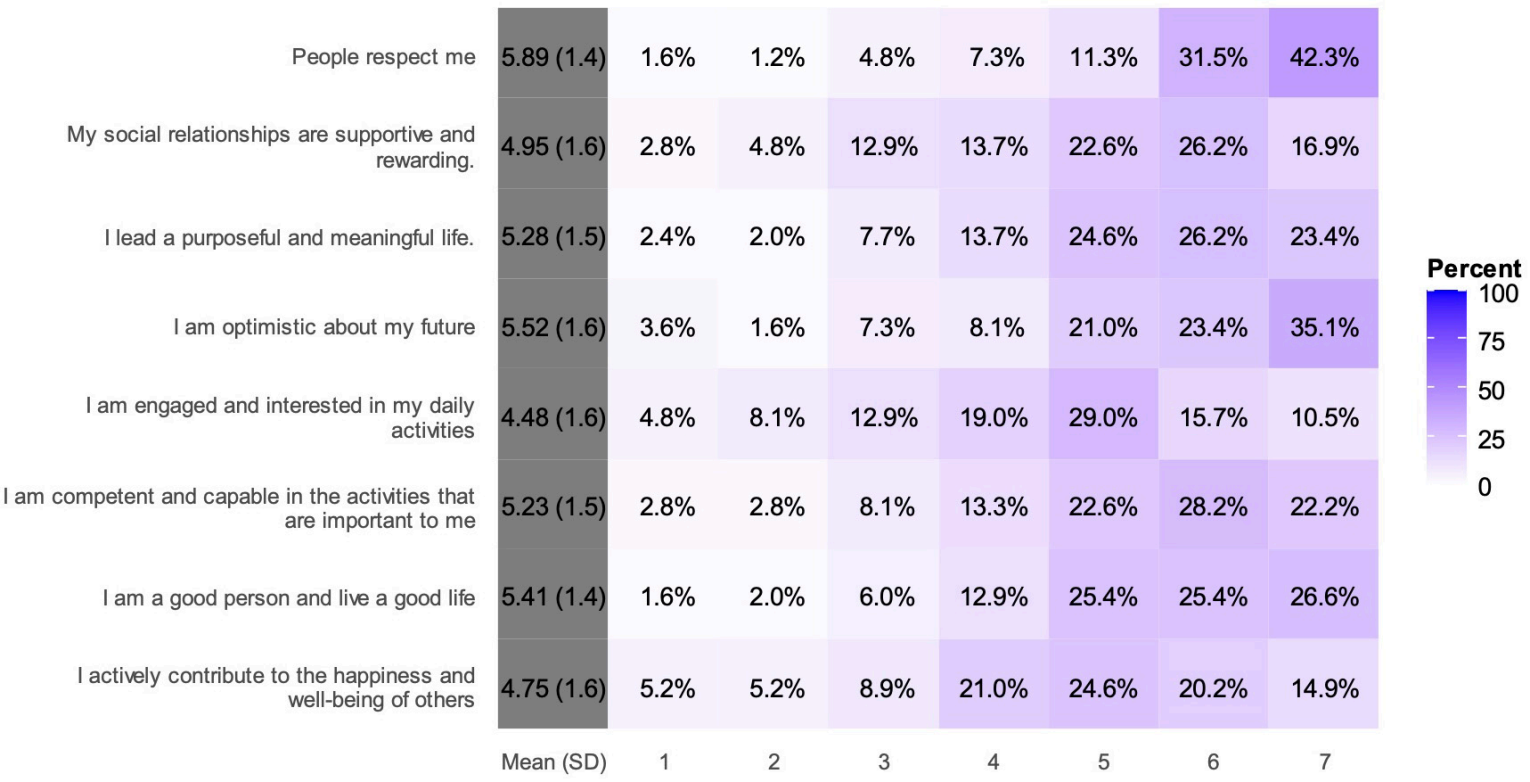
Distribution of responses for the Flourishing Scale items.

Mean scores on the Flourishing Scale items ranged from 4.48 (engagement in daily activities) to 5.89 (feeling respected by others). Figure 2 illustrates the distribution of responses from each Flourishing Scale items. Optimism about the future also ranked high, with 58.5% scoring 6 or 7. Half of the respondents (50.8%) reported leading purposeful and meaningful lives, while a similar proportion (50.4%) felt competent in activities important to them. A positive self-image was evident, with 52% scoring 6 or 7 on being a good person and living a good life. Social relationships were supportive for many, with 43.1% scoring 6 or 7. Engagement in daily activities scored lowest, with a mean of 4.48 and only 26.2% scoring 6 or 7. Contribution to others’ well-being showed mixed results, with 35.1% scoring 6 or 7. The scale ranged from 1 (strongly disagree) to 7 (strongly agree), with darker purple in the image indicating higher percentages of responses. Each item’s mean and standard deviation were provided.

### 3.2 Correlation between PSQI, Flourishing Scale, and GPA

**FIGURE 3:**
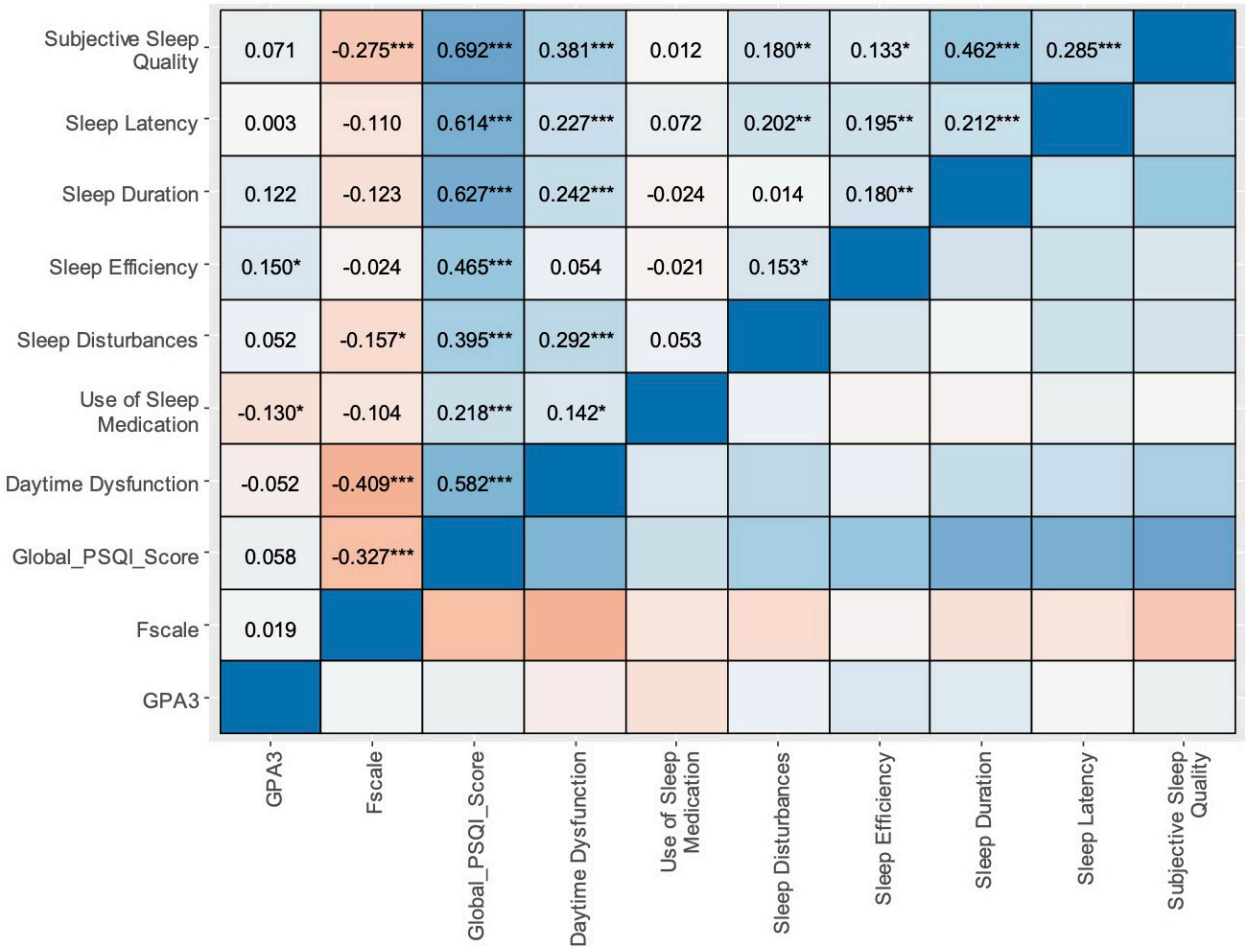
Correlation between sleep quality components, flourishing, and academic performance. This figure presents a correlation matrix heatmap illustrating the relationships between PSQI components, global PSQI score, Flourishing Scale (F scale), and GPA3. The color intensity represents the strength of correlations, with blue indicating positive and red indicating negative correlations. Darker shades signify stronger associations. Asterisks denote significance levels (*p < 0.05, **p < 0.01, ***p < 0.001).

Figure 3 presents the correlation between sleep quality components, flourishing, and academic performance. Sleep efficiency showed a very weak positive correlation with GPA (*r* = 0.150, *p* < 0.05). Conversely, use of sleep medication had a very weak negative correlation with GPA (*r* = −0.130, *p* < 0.05). Other sleep components and the Global PSQI Score did not show significant correlations with GPA (all *p* > 0.05). The Flourishing Scale showed significant negative correlations with several PSQI components and the Global PSQI Score. The strongest negative correlation was with Daytime Dysfunction (*r* = −0.409, *p* < 0.001), followed by the Global PSQI Score (*r* = −0.327, *p* < 0.001) and Subjective Sleep Quality (*r* = −0.275, *p* < 0.001). Among PSQI components, Subjective Sleep Quality showed moderate positive correlations with Sleep Latency (*r* = 0.285, *p* < 0.001) and Sleep Duration (*r* = 0.462, *p* < 0.001). Daytime Dysfunction correlated moderately with several other components (e.g., with Sleep Disturbances, *r* = 0.292, *p* < 0.001).

### 3.3 Ordinal logistic regression

The proportional odds assumption was verified using the nominal test (*p* = 0.41), confirming the appropriateness of the ordinal logistic regression model. Among the PSQI components, sleep duration emerged as a significant predictor. Results showed that students who slept longer were less likely to fall into lower GPA categories (OR = 0.72, 95% CI [0.55, 0.93], *p* = 0.01). This underscores the importance of adequate sleep for academic success. Students who used sleep medication more frequently had higher odds (OR = 1.48, 5% CI [1.03, 2.12], *p* = 0.035) of being in a lower GPA category. Daytime dysfunction, while not statistically significant (*p* = 0.066), showed a trend toward being associated with lower GPA. Male gender was associated with higher odds of being in a lower GPA category (OR = 4.31, 95% CI [1.96, 9.48], *p* < 0.001). However, this association may reflect contextual or institutional factors, as gender distribution differed substantially by university (Table 2). The final model explained 19.3 of the variance in GPA (Nagelkerke R^2^ = 0.193).

**TABLE 2:** Ordinal logistic regression analysis results.

	GPA		
Predictors	Odds ratios	95% CI	*p*
Daytime dysfunction	1.37	0.98–1.92	**0.066**
Sleep duration	0.72	0.55–0.93	**0.011**
Use of sleep medication	1.48	1.03–2.12	**0.035**
University: B vs. A	0.60	0.32–1.14	0.122
Level (1 level increase)	1.08	0.98–1.20	0.123
Gender: male vs. female	4.31	1.96–9.48	**<0.001**
Observations	248		
R^2^ Nagelkerke	0.193		

The Flourishing Scale score was not included, as it was not significantly associated with GPA when univariate analysis was used. Bold values indicate significance.

## 4 Discussion

### 4.1 Summary of key findings

The current study investigated the complex interrelationship between sleep quality, psychological well-being, and academic performance in Saudi university students. Results indicated that daytime dysfunction and sleep disturbances are pressing issues, whereby 93.2% of respondents reported daytime dysfunction to some degree and 14.5% were exposed to extreme levels of dysfunction. Additionally, sleep disturbances have been nearly general, affecting 96% of the sample. These findings support the extant literature, which indicates that sleep disorders among the student population are detrimental to cognitive functioning, particularly in the challenging and demanding academic environment (26, 27). The severe daytime dysfunction, as reported by a significant proportion of the participants, may interfere with their cognitive functions, such as attention and memory mechanisms, which are essential for good academic performance. Indeed, previous research documented that poor daytime functioning, such as systematic fatigue and reduced cognitive performance, is linked to comparatively academic performance (28).

### 4.2 Interpretation and comparison with previous studies

While sleep efficiency was high in most respondents, with 72.8% of the total respondents reporting good sleep efficiency, it did not help to improve the alarming trends in sleep duration. Only 25% reported optimal sleep duration, while the rest (40%) had poor to very poor sleep duration. This constitutes a significant difference, as adequate sleep duration is relevant for memory consolidation and cognitive performance, both vital elements in academic success (29, 30). These findings are consistent with a previous study that reported that students with shorter sleep durations tended to perform worse academically, which could be explained by the fact that inadequate sleep disrupts essential cognitive processes such as attention, memory consolidation, and problem-solving abilities. Such cognitive deficiency affects the ability of the students to retain information and effectively engage in academic tasks, leading to lower grades and overall academic underachievement (31). Poor sleep quality, similarly demonstrated in the form of shorter sleep durations and an increase in sleep disturbance, was associated with a reduction in cognitive performance, which is an essential component for academic success. The authors reported that students who slept less were more likely to experience daytime sleepiness, fatigue, and impaired concentration, which affect and hinder learning and exam performance (32). Both studies reinforced the notion that insufficient sleep not only affects immediate cognitive functions but also has negative cumulative effects on students’ academic performance.

Mixed results were observed for sleep latency, with approximately one-half (55.7%) of respondents reporting good to fair latency, while a significant 44.2% struggled with poor latency. Prolonged sleep latency is commonly associated with increased stress and anxiety, which are common among student populations due to academic pressures (33, 34). This suggests that stress-reduction interventions may improve sleep latency, sleep quality and academic performance. Interestingly, subjective sleep quality was relatively positive, with two-thirds of participants rating their sleep as good to fair. However, this contrasts with the objective measures of sleep duration and latency, where a substantial proportion of students experienced poor outcomes. This disparity between subjective and objective sleep quality was reported in previous studies, where students often overestimated their sleep quality despite measurable disturbances (35).

The low prevalence of sleep medication use among students in the current study (17.7%) reflects the broader trends observed in similar populations where young adults, particularly university students, generally show lower rates of prescription sleep aid usage compared to older adults. These findings are consistent with other studies examining sleep quality and sleep medication use in student populations which reported low rates of sleep medication use among medical students in Saudi Arabia (35). Similarly, Wong and co-authors reported low rates of sleep medication use among university students in Hong Kong. They attributed this to the common perception that such medications are only necessary for more severe or chronic sleep issues, which are less common in younger populations (33). A study among medical students in Slovenia showed a similarly low prevalence of sleep medication use (10.4), suggesting that students often avoid medical treatment despite experiencing sleep disturbances (36). The causes of the low use of sleep medications was not investigated in the current study but it might be attributed to the tendency of younger individuals to rely on non-pharmacological methods to manage sleep disturbances, the students concerns about the potential side effects of medications, or a reluctance to become dependent on sleep aids at a young age. The previous findings suggest a complex relationship between sleep quality, psychological well-being, and academic performance and underscore the need for universities to provide better resources for sleep hygiene education and support, as well as stress reduction programs, to mitigate the negative impact of poor sleep on academic performance.

The FS results revealed general positive self-perceptions among respondents. These findings align with previous research emphasizing the role of positive psychological traits in well-being and academic outcomes. Feeling respected, which had the highest mean score, aligns with studies linking social acceptance and respect to higher life satisfaction and engagement (37, 38). Optimism about the future, ranking high in this study, reflects findings in flourishing research that optimism contributes significantly to both mental health and academic performance. Diener et al. noted the strong associations between self-esteem, purpose, and optimism as critical components of psychological flourishing (22). In addition, Datu highlighted that flourishing positively predicted self-reported academic achievement and emotional engagement, suggesting that students who feel respected and optimistic are more likely to excel academically and socially (38, 39).

On the other hand, other areas of the FS indicated room for improvement. For instance, engagement in daily activities scored the lowest, with only 26.2% of respondents rating it highly. This is concerning, as low engagement often predicts academic underperformance and life dissatisfaction. Van Zyl and Rothmann found that flourishing students exhibit higher levels of positive affect, life satisfaction, and engagement, while those with low levels of flourishing tend to struggle academically (40). Additionally, the relatively low score for contributions to others’ well-being (35.1%) suggests that while students may feel positive about themselves, they may focus less on prosocial behaviors, which research suggests are crucial for both social and academic success. Howell et al. further support this, showing that engagement is strongly associated with flourishing, academic achievement, and well-being (27).

### 4.3 Practical implications

The relation between flourishing, sleep, and academic performance necessitates further investigation and theoretical consideration. Flourishing, with its positive emotional and functioning aspects, may act as a shield or a buffer against the negative effects of poor sleep on academic outcomes. However, the lack of a significant relation between flourishing and GPA in the current study, associated with the correlation between flourishing and sleep quality, suggests that this buffering or shield effect, if exist, maybe limited or that other mediating factors might be involved. The bidirectional relation between flourishing and sleep made it difficult to decide whether poor sleep reduced flourishing, affecting its emotional regulation and cognitive function, or low flourishing led to poor sleep behaviors through reduced motivation for self-care. The cross-sectional design of the study limited the ability to draw definitive conclusions regarding causality. However, the strong negative correlation between flourishing and daytime dysfunction suggests that the sleep-flourishing relationship may operate through daytime impairment rather than nighttime sleep quality.

A small positive correlation was observed between sleep efficiency and GPA, suggesting that a higher proportion of time spent asleep while in bed–an essential component for restorative sleep–maybe associated with better academic performance. Enhanced sleep efficiency can improve cognitive functions such as memory consolidation, attention, and executive functioning, which are critical for academic success (41). Consistent with previous research, higher sleep quality has been linked to improved academic outcomes (42, 43). Conversely, the use of sleep medication was negatively correlated with GPA. This suggests that students who frequently use sleep medications are more likely to have lower GPAs. The reliance on sleep medication may indicate underlying sleep disturbances or disorders that can impair cognitive performance and daytime functioning (44). Additionally, certain sleep medications may have side effects such as daytime drowsiness and reduced concentration, which can adversely affect academic performance (45).

The current findings are consistent with emerging research investigating physical therapy students, where sleep quality substantially influenced the students’ academic performance (20). While others reported an association between mental and physical health behaviors and academic outcomes in physical therapy doctoral students (18). Similarly, sleep duration was a significant predictor of academic performance in physical therapy students, aligning with our observation that sleep duration predicated GPA category (17). However, methodological differences across these studies, including variations in sample sizes, geographic contexts, and measurement instruments, limit direct comparisons with the current findings. The Flourishing Scale exhibited significant negative correlations with several PSQI components, including Daytime Dysfunction, the Global PSQI Score, and Subjective Sleep Quality. These findings suggest that higher levels of psychological well-being are associated with better sleep quality. Psychological well-being, as measured by the Flourishing Scale, encompasses aspects such as positive relationships, self-esteem, and a sense of purpose, which can contribute to healthier sleep patterns (46). Daytime dysfunction, which showed the strongest negative correlation with flourishing, reflects difficulties in staying awake and maintaining alertness during the day. High levels of daytime dysfunction can impede daily activities and reduce overall well-being (47). The substantial negative correlation implies that students experiencing less daytime dysfunction tend to report higher psychological well-being, aligning with literature that underscores the bidirectional relationship between sleep quality and mental health (48, 49). While the study findings aligned with several previous studies, these results should be interpreted cautiously given the cross-sectional nature of the available evidence. Many prior studies in this area also relied on self-reported measures, which may introduce similar biases across the literature (42).

Gender also played a significant role in predicting academic performance, with male students having substantially higher odds of being in a lower GPA category compared to female students. This pronounced gender disparity highlights the need for targeted support and interventions to address the unique challenges faced by male students in academic settings (50). Alshahrani et al., discovered that the university female students were able to modify their sleep patterns between summer vacation and the first 4 weeks of the academic year and that their sleeping hours were shorter during the academic period than during the summer. This raises concerns about how well equipped female students are to adjust to these challenges compared to their male counterparts if they have a changing sleeping pattern as well (51).

### 4.4 Strengths and limitations

The study strengths include the use of validated instruments (PSQI and Flourishing Scale), an adequate sample size for statistical analysis, and the inclusion of students from two different universities to enhance regional generalizability. However, the observed correlations were generally small to moderate in magnitude. Given the cross-sectional design, causal inferences cannot be drawn, and the findings should be therefore interpreted as preliminary. The study was limited to universities in Riyadh, Saudi Arabia; although these institutions attract students from multiple governorate, the findings may not be generalized to all physiotherapy students nationwide. The response rate of 76.8 may have introduced selection bias, as students with greater interest in sleep or well-being may have been more likely to participate. In addition, the complete confounding of gender with university affiliation prevents clear separation of gender effects from institutional effects. Consequently, the observed gender-GPA association should be interpreted as an adjusted statistical association rather than a causal or modifiable factor. Finally, GPA was self-reported and may be subject to recall or social desirability bias, although anonymity was emphasized to mitigate this concern.

### 4.5 Future directions and conclusion

The current study examined the relationship between sleep quality, flourishing, and academic performance among physical therapy students in Riyadh. The findings indicate that a large proportion of students experience poor sleep quality despite reporting relatively high levels of flourishing. Sleep disturbances were prevalent across both universities and differed significantly by gender. In addition, a negative association was observed between global PSQI scores and flourishing. Notably, sleep quality and flourishing were not significantly associated with GPA, suggesting that academic performance may be influenced by additional academic, institutional, or psychosocial factors. These findings highlight the importance of addressing sleep health within a university setting. However, more studies are needed to examine whether sleep hygiene interventions implemented through university counseling centers are associated with improvement in sleep quality and overall well-being. Physiotherapy students should be educated about the importance of sleep for mental health and daily functioning. Future research should extend to other regions and include students from additional health professions to enhance external validity. Longitudinal and interventional studies are recommended to clarify causal pathways between sleep quality, flourishing, and academic performance. Further studies should also consider additional psychosocial and behavioral variables that may influence academic outcomes.

## Data Availability

The data supporting the findings of this study are not publicly available due to institutional policies that restrict data sharing. Requests to access the datasets should be directed to AMAttia@pnu.edu.sa.
